# Modifying gene expression through passive forces

**DOI:** 10.7554/eLife.107575

**Published:** 2025-06-30

**Authors:** Sarah Hallstein, Julian König

**Affiliations:** 1 Gene Regulation in Evolution, Institute for Molecular Biology gGmbH Mainz Germany; 2 Theodor-Boveri-Institute, University of Würzburg Würzburg Germany

**Keywords:** m6A, epitranscriptome, dynamics, model, RNA-seq, Human, Mouse

## Abstract

The mRNA metabolism passively shapes the levels of an mRNA modification called m6A within a steady-state cell and upon stress.

**Related research article** Dierks D, Shachar R, Nir R, Garcia-Campos MA, Uzonyi A, Wiener D, Toth U, Rossmanith W, Lasman L, Slobodin B, Hanna JH, Antebi Y, Scherz-Shouval R, Schwartz S. 2025. Passive shaping of intra- and intercellular m6A dynamics via mRNA metabolism. *eLife* **13**:RP100448. doi: 10.7554/eLife.100448.

Each cell type has a different set of active genes that allow it to carry out a distinct role. Cells need to be able to change which genes are expressed efficiently in response to different stimuli. To do so, they use various ways to control gene expression to increase or decrease the production of specific gene products, such as proteins. One way to change gene expression is by modifying messenger RNA, or mRNA. Such changes are thought to affect nearly every step of the RNA lifecycle and are extremely versatile. A single modification could reduce or even terminate protein production or alter the identity of an amino acid sequence. Such mRNA modifications are thus a powerful way to modify gene expression and influence many biological processes.

One of the most abundant changes to mRNAs is the methylation of the *N*6 position of adenosine. This modification – which is known as *N*6-methyladenosine (m6A) – influences crucial steps in mRNA processing, such as mRNA stability, and acts as a strong signal for mRNA degradation in the cytoplasm. Different writer and eraser proteins add or remove this modification, thereby actively shaping the levels of m6A (He and He, 2021).

Research has mainly focused on the active mechanisms that add or remove this mark at specific sites to change: various writer and eraser proteins act in the nucleus to add and remove m6A marks as RNA is being made, while reader proteins trigger its degradation in the cytoplasm.

Previous research has suggested that m6A is highly conserved between different species and ‘hard-coded’ in the RNA, i.e., it is only added at pre-determined sites. However, m6A levels have been reported to change in different cellular compartments and across different external stimuli, such as stress responses. How such compartmental specificity can be achieved has so far been unclear.

Now, in eLife, Schraga Schwartz and colleagues – including David Dierks as first author – report that m6A dynamics are caused by semi-passive regulation (Dierks et al., 2025). The researchers, who are based at the Weizmann Institute of Science, the University of Washington and the Medical University of Vienna, developed a mathematical model, m6ADyn, to predict how key steps of the mRNA life cycle and m6A levels influence each other.

Specifically, they explored the interplay between m6A levels, mRNA decay in the cytoplasm mediated by m6A, and the relative distribution of mRNAs between the nucleus and the cytoplasm (Figure 1). They further expanded their model to mRNA transcription and m6A deposition.

**Figure 1.**
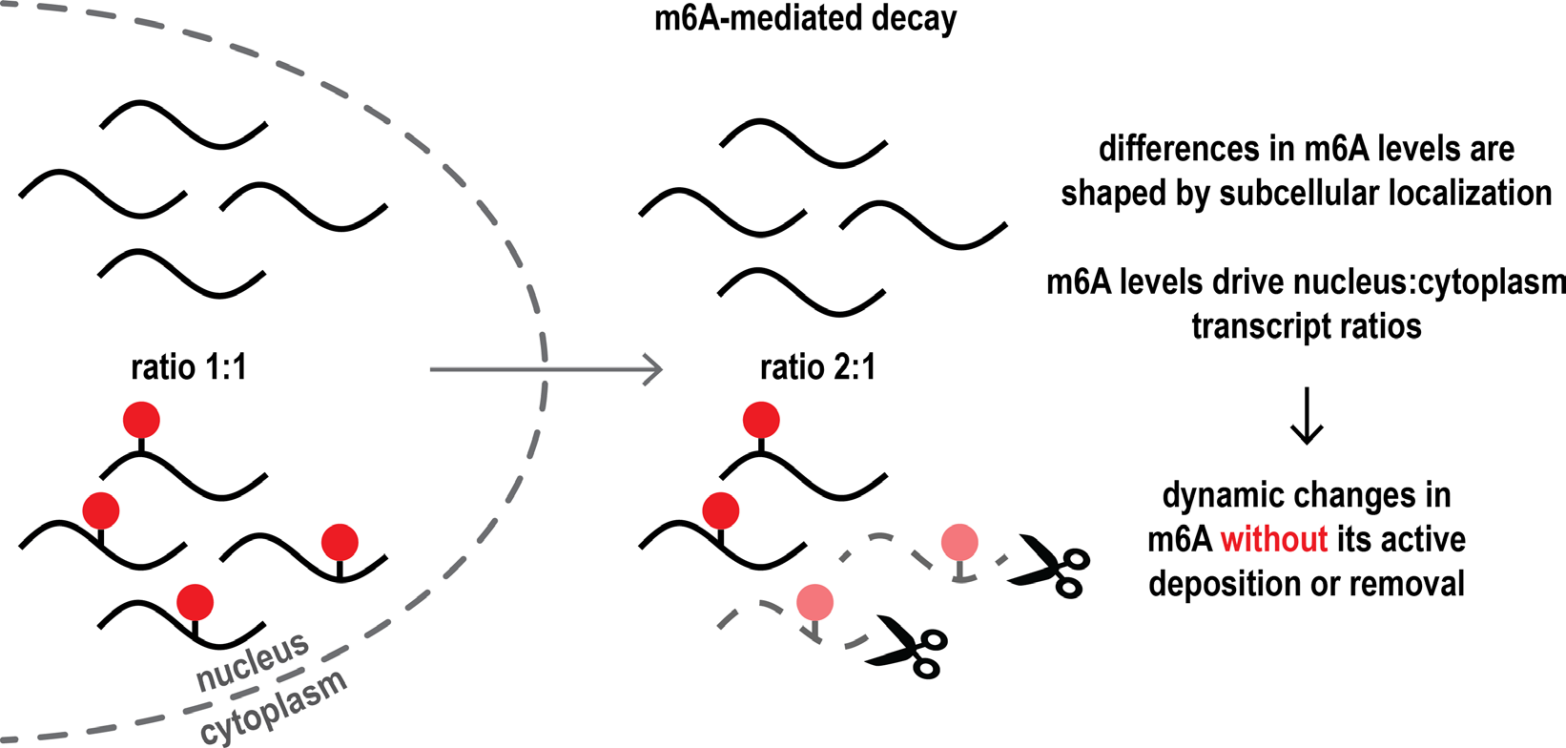
The interplay between m6A-mediated mRNA decay in the cytoplasm and subcellular mRNA distribution passively shapes m6A dynamics in cells. In the cell nucleus (left), the ratio of mRNA transcripts (black lines) with or without m6A chemical modifications (red dots) is even (exemplified as 1:1 ratio). However, once mRNA is exported into the cytoplasm (right), m6A-modified transcripts are degraded more frequently (scissors), resulting in an altered ratio of non-modified and modified mRNA in the cytoplasm. This explains how there can be dynamic changes in the levels of m6A across the nucleus and the cytoplasm without the need for m6A marks to be actively added to or removed from mRNA transcripts. Subcellular localization thus shapes m6A levels and influences the distribution and levels of mRNA.

The key assumption of the model was that because m6A promotes mRNA decay in a specific compartment and at a specific time in the RNA lifecycle, its levels are changed semi-passively: m6A marks lead to degradation in the cytoplasm, and as the mRNA is degraded, the m6A marks are also lost in this compartment. This would suggest that mRNA decay and m6A levels across different cellular compartments are intricately coupled. That m6A-mediated mRNA decay works most efficiently in the cytoplasm has also been supported by other experimental studies (Zhou et al., 2024; Linder et al., 2025; Murakami et al., 2025).

Dierks et al. found that a destabilizing effect (i.e. the increased degradation of mRNA and thus m6A) in just one compartment is sufficient to cause dynamic changes in m6A levels between the nucleus and the cytoplasm without the need for active addition or removal of m6A. The researchers then experimentally tested key m6ADyn predictions in human and mouse cell lines by measuring mRNA levels and estimating the stability of the mRNA (Dierks et al., 2021).

They challenged their model under stable conditions and focused on intracellular m6A distribution and mRNA stability. They detected higher m6A levels for nuclear transcripts and found that, in the cytoplasm, transcripts with an m6A mark were removed faster than the ones without a mark. These observations are consistent with the key assumption that the degradation of m6A-containing transcripts mainly occurs in the cytoplasm, thereby passively shaping the nuclear-cytoplasmic distribution of mRNAs. These findings also suggest that differences in m6A levels can be explained by the subcellular localization and stability of transcripts,i.e., in the cytoplasm, where more mRNA degradation occurs.

Next, they applied their model to scenarios mimicking disruptions of cellular mRNA processes and stress. Consistent with their model’s predictions, inhibiting transcription, m6A-dependent cytoplasmic decay, or exposure to heat shock led to changes in m6A levels. Further analysis revealed that heat stress affected m6A levels predominantly on nuclear-enriched RNA.

Collectively, this study provides new insights into m6A dynamics. Consistent with current research (Anders et al., 2018; Liu et al., 2023), the results support that changes in m6A levels are often less pronounced than initially expected. Dierks et al. approached the complexity of the system with a simple mathematical model and a few key assumptions. They conclude that the mRNA metabolism passively influences and shapes m6A levels within a steady-state cell and upon stress. In more detail, they report that m6A-mediated degradation in the cytoplasm plays an essential role in m6A dynamics.

Importantly, to date, changes in m6A levels have mostly been explained by active processes. For example, differences in m6A between the nucleus and cytoplasm were seen as indicative of m6A impacting nuclear export (Edens et al., 2019; Hsu et al., 2019). In contrast, this study implies a central role of passive mechanisms in shaping the m6A landscape, rather than active ones. This gives rise to a new question: how do active mechanisms (writers and erasers) and passive (the mRNA metabolism) influence each other? By including additional parameters, such as further functions of m6A or transcript-specific predictions, m6ADyn has the potential to capture the full complexity of m6A regulation in the future.
